# Frontiers in Antimicrobial Materials

**DOI:** 10.3390/ijms23148047

**Published:** 2022-07-21

**Authors:** Ángel Serrano-Aroca, Murtaza M. Tambuwala, Martin Birkett

**Affiliations:** 1 Biomaterials and Bioengineering Lab, Centro de Investigación Traslacional San Alberto Magno, Universidad Católica de Valencia San Vicente Mártir, 46001 Valencia, Spain; 2 School of Pharmacy and Pharmaceutical Sciences, Ulster University, Cromore Road, Colerine BT52 2FB, UK; m.tambuwala@ulster.ac.uk; 3 Department of Mechanical and Construction Engineering, Northumbria University, Ellison Place, Newcastle upon Tyne NE1 8ST, UK; martin.birkett@northumbria.ac.uk

The aim of this Special Edition is to highlight the exponential work performed in the field of antimicrobial material research from the beginning of the current COVID-19 pandemic. This issue provides new insights into antimicrobial solutions that can prevent viral and bacterial infections, particularly against SARS-CoV-2, which causes COVID-19 disease. Thus, pathogens have been shown to be capable of causing disastrous effects on global human health and the economy. Antimicrobial resistance is another global health threat that is increasing at an alarming rate and has caused more than one million deaths in 2021. Viral and bacterial transmission can occur through material contact and aerosols. Therefore, the development of new advanced antimicrobial materials and coatings capable of preventing viral and bacterial transmission is becoming essential to keep humans safe from emerging infectious microorganisms. In this context, due to the increasing international interest in metal-based antimicrobial coatings to control the spread of bacteria, fungi, and viruses, Birkett et al. presented a review on the recent advances in metal-based antimicrobial coatings for high-touch surface applications, such as hand rails, door plates, and water fittings on public transport, and in healthcare, care homes and leisure settings, as well as personal protective equipment commonly used in hospitals [1]. The COVID-19 pandemic has highlighted the major role that antimicrobial coatings can play in controlling the spread of deadly viruses, such as SARS-CoV-2. In this review, three discrete microorganism-killing phenomena of contact-killing surfaces, nanoprotrusions, and superhydrophobic surfaces are presented. The antimicrobial performance of antimicrobial metals, such as Cu, Ag, and Zn, are presented, along with the effects of combining them with TiO_2_ to create a next generation of antimicrobial coating materials. In this regard, Pereira et al. present a recent and comprehensive review on the state-of-the-art in the use of metals, as well as their mechanisms, to fight different clinically relevant microorganisms, such as viruses, bacteria, and fungi [2]. This review demonstrates that Cu is among the metals with the best antimicrobial properties against several microorganisms and how antiwetting capacity is important to enhance the biocidal activity of metal surfaces or metal-coated surfaces. Additive manufacturing (AM) has been shown to be an alternative technology to develop antiviral materials. Thus, Arjunan et al. showed a novel use of Laser Powder Bed Fusion (LPBF) to produce a 3D printed porous Cobalt–Chromium–Molybdenum (Co–Cr–Mo) superalloy with potent antiviral activity (100% viral inactivation in 30 min) against a surrogate of SARS-CoV-2 [3]. The paper shows a model that allows us to tailor on-demand porosity without the need for complex geometry data. The material significantly outperforms the viral inactivation time of copper and silver. This study provides a porous substrate that can be used to combat SARS-CoV-2 viral spread. Therefore, the proposed AM methodology can be adopted to conceive functional antimicrobial materials that can be fabricated close to the point of care. On the other hand, Takayama et al. propose the use of a non-woven fabric functionalized with a biobased product, cranberry extract, to combat COVID-19 and multidrug-resistant bacteria [4]. These advanced fabrics can be used for infection prevention clothing, such as face masks, caps, scrubs, shirts, trousers, disposable gowns, overalls, hoods, aprons and shoe covers, as protective tools against viral and bacterial infections, including methicillin-resistant *Staphylococcus aureus* (MRSA) and *epidermidis* (MRSE). The null toxicity of these antimicrobial fabrics was demonstrated in vivo using the *Caenorhabditis elegans* model. Functionalized anti-microbial material developed by Luca et al. provides proof of concept for the first time for the use of an ApoB-derived natural anti-microbial peptide to functionalize Polydimethylsiloxane (PDMS) to produce a stable, antimicrobial, and biocompatible material for medical devices, such as catheters. They have demonstrated that PDMS loaded with ApoBL Pro peptide did not have any adverse effect on the viability of eukaryotic cells, indicating that it is biocompatible and safe for human use [5]. The development and use of anti-microbial materials could result in a reduction in catheter-related bacterial infections in patients. There is also growing interest in food packaging materials that can play an active role in preservation. Ordon et al. investigated polyethylene films containing plant extracts in the polymer matrix as antibacterial and antiviral materials. They found that low-density polyethylene films covered with active coatings containing mixtures of rosemary, raspberry and pomegranate CO_2_ extracts were active against selected Gram-positive and Gram-negative bacteria strains and also offered antiviral activity against a surrogate of SARS-CoV-2 [6]. The modified polyethylene CO_2_ extracts showed good antimicrobial performance and the potential to be applied as a functional packaging material to extend the shelf life of food products.
